# Remote rhythm monitoring using a photoplethysmography smartphone application after cardioversion for atrial fibrillation

**DOI:** 10.1093/ehjdh/ztae028

**Published:** 2024-04-15

**Authors:** Peter Calvert, Mark T Mills, Kelly Howarth, Sini Aykara, Lindsay Lunt, Helen Brewer, David Green, Janet Green, Simon Moore, Jude Almutawa, Dominik Linz, Gregory Y H Lip, Derick Todd, Dhiraj Gupta

**Affiliations:** Liverpool Centre for Cardiovascular Science at University of Liverpool, Liverpool John Moores University and Liverpool Heart & Chest Hospital, Thomas Drive, Liverpool L14 3PE, UK; Department of Cardiology, Liverpool Heart & Chest Hospital NHS Foundation Trust, Thomas Drive, Liverpool L14 3PE, UK; Liverpool Centre for Cardiovascular Science at University of Liverpool, Liverpool John Moores University and Liverpool Heart & Chest Hospital, Thomas Drive, Liverpool L14 3PE, UK; Department of Cardiology, Liverpool Heart & Chest Hospital NHS Foundation Trust, Thomas Drive, Liverpool L14 3PE, UK; Department of Cardiology, Liverpool Heart & Chest Hospital NHS Foundation Trust, Thomas Drive, Liverpool L14 3PE, UK; Department of Cardiology, Liverpool Heart & Chest Hospital NHS Foundation Trust, Thomas Drive, Liverpool L14 3PE, UK; Department of Cardiology, Liverpool Heart & Chest Hospital NHS Foundation Trust, Thomas Drive, Liverpool L14 3PE, UK; Department of Cardiology, Liverpool Heart & Chest Hospital NHS Foundation Trust, Thomas Drive, Liverpool L14 3PE, UK; Department of Cardiology, Liverpool Heart & Chest Hospital NHS Foundation Trust, Thomas Drive, Liverpool L14 3PE, UK; Department of Cardiology, Liverpool Heart & Chest Hospital NHS Foundation Trust, Thomas Drive, Liverpool L14 3PE, UK; Department of Cardiology, Liverpool Heart & Chest Hospital NHS Foundation Trust, Thomas Drive, Liverpool L14 3PE, UK; Department of Cardiology, Liverpool Heart & Chest Hospital NHS Foundation Trust, Thomas Drive, Liverpool L14 3PE, UK; Department of Cardiology, Maastricht University Medical Center and Cardiovascular Research Institute Maastricht, Maastricht, The Netherlands; Department of Biomedical Sciences, Faculty of Health and Medical Sciences, University of Copenhagen, Copenhagen, Denmark; Liverpool Centre for Cardiovascular Science at University of Liverpool, Liverpool John Moores University and Liverpool Heart & Chest Hospital, Thomas Drive, Liverpool L14 3PE, UK; Department of Cardiology, Liverpool Heart & Chest Hospital NHS Foundation Trust, Thomas Drive, Liverpool L14 3PE, UK; Danish Center for Health Services Research, Department of Clinical Medicine, Aalborg University, Aalborg, Denmark; Department of Cardiology, Liverpool Heart & Chest Hospital NHS Foundation Trust, Thomas Drive, Liverpool L14 3PE, UK; Liverpool Centre for Cardiovascular Science at University of Liverpool, Liverpool John Moores University and Liverpool Heart & Chest Hospital, Thomas Drive, Liverpool L14 3PE, UK; Department of Cardiology, Liverpool Heart & Chest Hospital NHS Foundation Trust, Thomas Drive, Liverpool L14 3PE, UK

**Keywords:** Atrial fibrillation, Cardioversion, Photoplethysmography, FibriCheck

## Abstract

**Aims:**

Direct current cardioversion (DCCV) is a commonly utilized rhythm control technique for atrial fibrillation. Follow-up typically comprises a hospital visit for 12-lead electrocardiogram (ECG) two weeks post-DCCV. We report the feasibility, costs, and environmental benefit of remote photoplethysmography (PPG) monitoring as an alternative.

**Methods and results:**

We retrospectively analysed DCCV cases at our centre from May 2020 to October 2022. Patients were stratified into those with remote PPG follow-up and those with traditional 12-lead ECG follow-up. Monitoring type was decided by the specialist nurse performing the DCCV at the time of the procedure after discussing with the patient and offering them both options if appropriate. Outcomes included the proportion of patients who underwent PPG monitoring, patient compliance and experience, and cost, travel, and environmental impact. Four hundred sixteen patients underwent 461 acutely successful DCCV procedures. Two hundred forty-six underwent PPG follow-up whilst 214 underwent ECG follow-up. Patient compliance was high (PPG 89.4% vs. ECG 89.8%; *P* > 0.999) and the majority of PPG users (90%) found the app easy to use. Sinus rhythm was maintained in 71.1% (PPG) and 64.7% (ECG) of patients (*P* = 0.161). Twenty-nine (11.8%) PPG patients subsequently required an ECG either due to non-compliance, technical failure, or inconclusive PPG readings. Despite this, mean healthcare costs (£47.91 vs. £135 per patient; *P* < 0.001) and median cost to the patient (£0 vs. £5.97; *P* < 0.001) were lower with PPG. Median travel time per patient (0 vs. 44 min; *P* < 0.001) and CO_2_ emissions (0 vs. 3.59 kg; *P* < 0.001) were also lower with PPG. No safety issues were identified.

**Conclusion:**

Remote PPG monitoring is a viable method of assessing for arrhythmia recurrence post-DCCV. This approach may save patients significant travel time, reduce environmental CO_2_ emission, and be cost saving in a publicly-funded healthcare system.

## Graphical Abstract

**Graphical Abstract:**
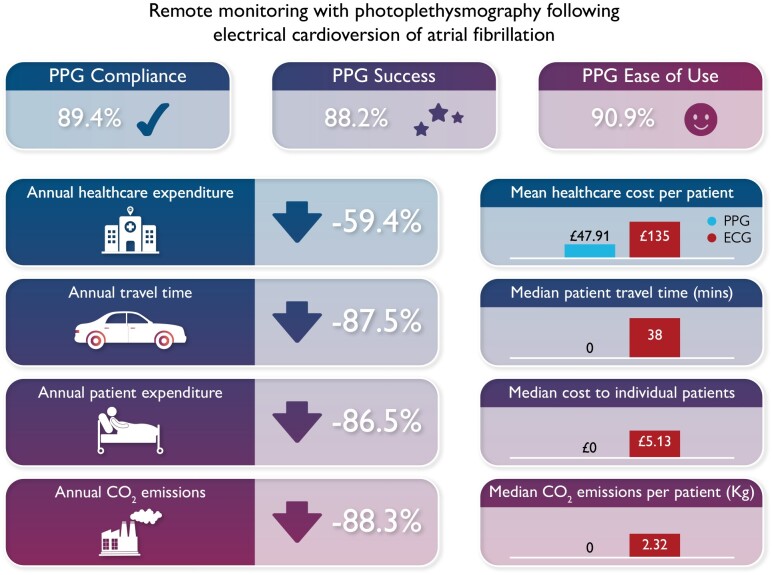
PPG, photoplethysmography. Compliance was defined as the percentage of patients who correctly registered and utilized the PPG app. Success was defined as the percentage of patients using PPG who had a successful rhythm diagnosis not requiring alternative rhythm assessment. Ease of use was defined as the percentage of patients enrolled in the prior TeleCheck-AF study who reported ‘strongly agree’ or ‘agree’ with the app being easy to use.

## Introduction

Atrial fibrillation (AF) is the most common sustained arrhythmia encountered in clinical practice. Whilst there are several facets involved in effective AF management, the decision to pursue rate or rhythm control is a major consideration.^1^

When pursuing a rhythm control strategy, a helpful starting point is to consider cardioversion, which may be achieved using direct current cardioversion (DCCV) or drugs. In appropriately selected patients, DCCV has high initial success rates and may substantially improve symptom burden from AF.^2^ Assessment of the patient in sinus rhythm allows symptom–rhythm correlation^3^ and onward decision making with regard to lifestyle improvements, antiarrhythmic medication, or invasive rhythm control management.

Early AF recurrence following DCCV is common.^4^ For this reason, it is common practice to monitor patients for recurrence in the short-term following DCCV. Whilst there is no fixed standard for this, our local practice up until mid-2020 was to recall the patient for a 12-lead electrocardiogram (ECG) two weeks following DCCV. This approach necessitates patients making a journey to and from the hospital with associated time, financial, and environmental costs. Recent advances in smartphone apps and wearables have allowed remote monitoring of heart rhythm thereby presenting an opportunity to overcome some of these limitations.

The first wave of the COVID-19 pandemic arrived in the UK in early 2020, with severe restrictions placed on patient visits to hospital for non-emergency indications. This had a significant impact on our arrhythmia service,^5^ necessitating a radical change in the way our DCCV service was managed.

**Figure 1:**
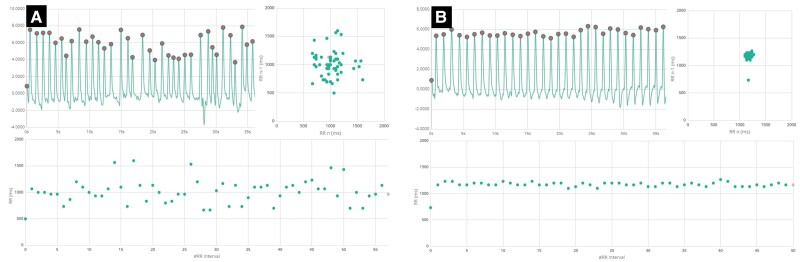
Examples of PPG output. (*A*) atrial fibrillation. (*B*) Sinus rhythm. Top left: PPG signals. Top right: Poincare plots. Bottom: Tachograms. Note the dispersion of RR intervals seen in atrial fibrillation versus sinus rhythm.

FibriCheck® (Qompium, Hasselt, Belgium) is an app that utilizes photoplethysmography (PPG) via the smartphone camera, along with an artificial intelligence (AI) algorithm, to distinguish AF from sinus rhythm. This approach has high sensitivity (96%) and specificity (97%),^6^ has been validated as highly accurate in clinical studies,^7,8^ and was utilized as part of a comprehensive telemedicine approach to AF management in the TeleCheck-AF study.^9–11^ Examples of AF and sinus rhythm detected by FibriCheck are shown in *Figure 1*. In April 2020, we had a series of remote meetings with manufacturers of FibriCheck, where our heart rhythm nurses (HRNs) and physicians were afforded training on the use of this app and interpretation of its findings. Physician interpretation of PPG findings has been shown to have similar accuracy to 12-lead ECG.^12^ We integrated FibriCheck into the necessary move towards a remote monitoring service.

The purpose of this study was to evaluate our remote rhythm monitoring service using this smartphone app compared with traditional ECG monitoring, which was still utilized during this time in patients for whom PPG monitoring was considered inappropriate (e.g. bradyarrhythmia, or a desire to check the QT-interval when starting antiarrhythmic drugs). We compared the broader impact upon patient convenience, travel times, environmental impact, and healthcare costs.

## Methods

### Data extraction, inclusion, and exclusion criteria

We retrospectively analysed consecutive patients attending our DCCV follow-up service between May 2020 (FibriCheck implementation) and October 2022. Patients with acutely unsuccessful DCCV were excluded. We stratified patients into those with FibriCheck follow-up (PPG cohort) and those with traditional 2-week 12-lead ECG follow-up (ECG cohort). The decision on which monitoring type to utilize was made by the cardiac specialist nurse who performed the DCCV at the time of the procedure, after discussion with the patient and offering them both options if appropriate. The study was approved by our local Research & Innovation Committee.

Patients undergoing alternative types of rhythm monitoring (e.g. utilizing their own smart wearable, or having implantable device interrogation) were excluded. Data were extracted from our electronic patient records. Data from the FibriCheck patient experience survey specific to our centre were also analysed. The full results of this survey have previously been published as part of the TeleCheck-AF study.^10^

### Follow-up

All patients were followed up with a phone call from the HRN approximately two weeks post-DCCV.

**Figure 2:**
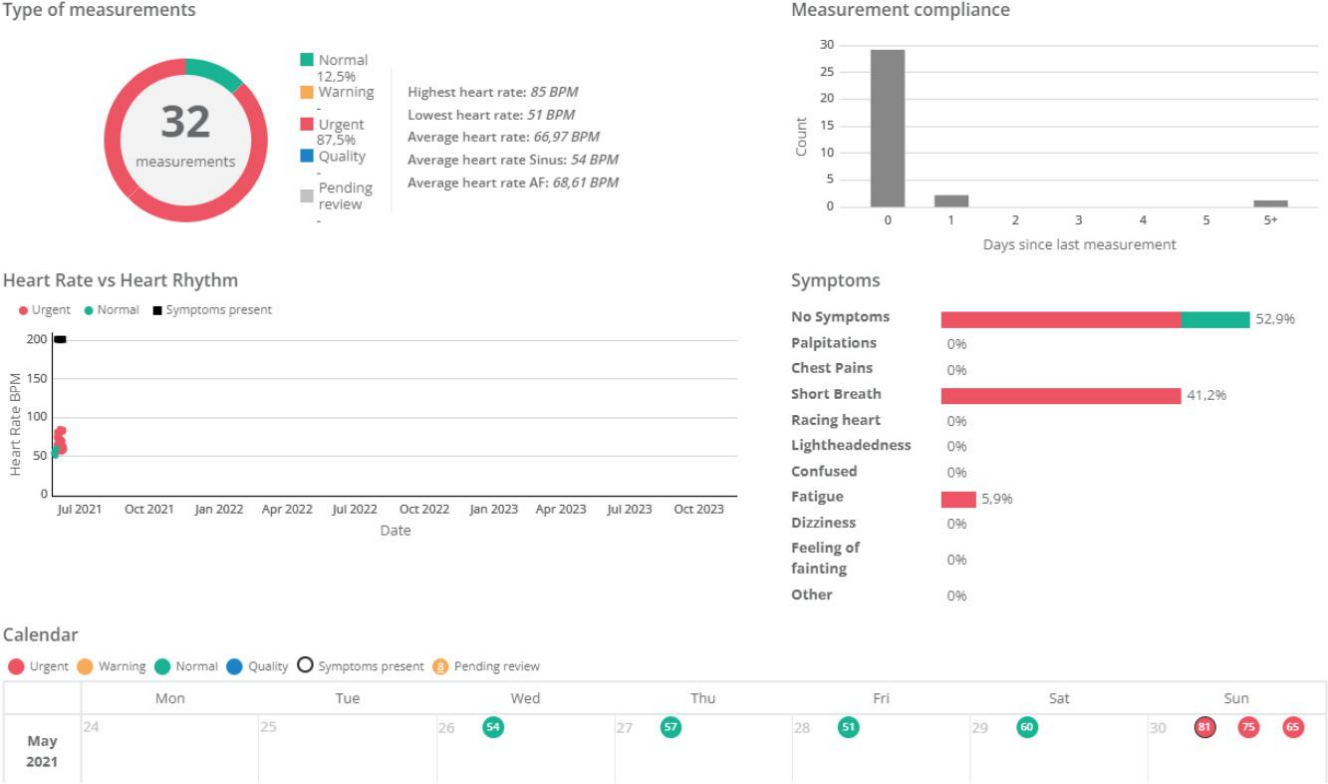
Example of the FibriCheck dashboard—in this case, the patient took multiple readings that demonstrated a recurrence of AF on Day 5. Measurements flagged as ‘urgent’ are due to AF detection by the algorithm.

Patients assigned to FibriCheck follow-up received an information sheet including a QR code for activation of FibriCheck on their smartphone. Patients then either self-installed the app or, upon request, were assisted by a nurse. After installation of the app, patients were connected to the FibriCheck telemedicine portal. Standard advice was to perform a single 1 min rhythm recording at 14 days post-DCCV, in order to coincide with the standard timing of ECG follow-up used prior to the pandemic. Patients were provided with a contact phone number for the HRN in case they required advice regarding symptoms or in utilizing the app. An example of the FibriCheck dashboard is shown in *Figure 2*.

Follow-up with a traditional 12-lead ECG at 2 weeks continued for those patients who either preferred not to use FibriCheck, were unable to use the app, or for clinical reasons, such as bradyarrhythmia post-DCCV, or a desire to check the QT-interval after starting antiarrhythmic drugs.

Patients assigned to PPG monitoring but who were unable to utilize the app were non-compliant (i.e. failed to register for the PPG app or did not utilize it despite registration), or where diagnostic uncertainty existed (e.g. due to low quality PPG traces), were asked to attend for a 12-lead ECG instead. For the purposes of this study, we counted these patients as part of the FibriCheck arm (intention to treat), even if they subsequently required an ECG.

### Cost and environmental analyses

The cost to the healthcare organization of a FibriCheck activation was £32, whilst the cost of a 12-lead ECG appointment was £135. Where patients assigned to FibriCheck subsequently needed an ECG, these costs were both applied. The total healthcare cost per patient was therefore the sum of FibriCheck activation costs and ECG appointment costs.

Costs to the patient were determined based upon their proximity to our centre, using their postcode. For patients living within the local area, we calculated their travel time and distance to our centre using Google Maps (normalized to a Monday morning at 10 a.m. for consistency). Travel costs were estimated based upon a standard 50 mpg diesel car at a fuel price of 162.65 pence per litre (the average UK diesel cost as of October 2023), which resulted in a cost of 15 p/mile. This was multiplied by the distance to the hospital, doubled to account for travel both ways. Parking costs of £3.60 (the minimum spend for up to 2 h at our centre) were also applied. For patients living outside of the local area, we applied a standard estimate of 5 miles travel to their local centre (10 miles in total for both directions) for an ECG, as well as the same parking cost. Patient travel times were estimated from Google Maps in the same fashion, and for those travelling to their local centre, we assumed an average speed of 20 mph, resulting in travel time of 30 min in total (10 miles at 20 mph). For those using FibriCheck successfully, no travel costs or times, nor parking costs, were applied.

Environmental impact was determined based upon the latest emissions data published in the UK.^13^ An average diesel car produces an estimated 26.76 kgCO_2_ emission over a distance of 118.06 miles, which gives ∼0.227 kgCO_2_/mile. We multiplied this figure by the number of miles travelled for each patient to determine overall CO_2_ emission in each arm.

For patients undergoing ECG follow-up, but who were non-compliant (i.e. did not attend for their ECG), healthcare costs for the missed appointment were applied, but patient travel costs and environmental costs were not applied, as the journey was not made. Non-compliance in the PPG arm only resulted in applied costs where a subsequent ECG appointment was made.

### Clinical outcomes

In order to quantify the uptake of PPG (which may be affected by clinician comfort with the technology), we compared the proportion of patients assigned to PPG vs. ECG monitoring. For those assigned to ECG, we assessed the reasons why PPG monitoring was not utilized. We also analysed rhythm at follow-up as well as patient compliance (defined as utilization of the FibriCheck app, or attendance for an ECG, respectively). Where patients were non-compliant, their follow-up rhythm was determined by any subsequent documentation (e.g. a subsequent ECG during clinic appointment). If this was not available due to a lack of subsequent follow-up, the patient was considered lost-to-follow-up.

Additionally, we analysed safety outcomes including bradyarrhythmia requiring intervention or hospitalization.

### Data analysis

Continuous variables are presented as mean ± standard deviation or median [IQR] depending upon statistical distribution and compared using *t*-tests or U-tests accordingly. Categorical variables are presented as counts and percentages and compared using Fisher’s exact test. A two-sided *P*-value < 0.05 was considered statistically significant. Statistical analysis was performed in R.

## Results

### Patient characteristics

Between 2 May 2020 and 31 October 2022, 416 patients underwent 461 acutely successful DCCV procedures (246 PPG follow-up, 215 ECG follow-up). Baseline patient characteristics are shown in *Table 1*. The majority of patients was male and around half had undergone a previous DCCV. The PPG group were younger on average (61.9 ± 9.5 vs. 66.4 ± 10.3 years; *P* < 0.001). Otherwise, comorbidities and medications were reasonably well-balanced between the groups.

**Table 1:**
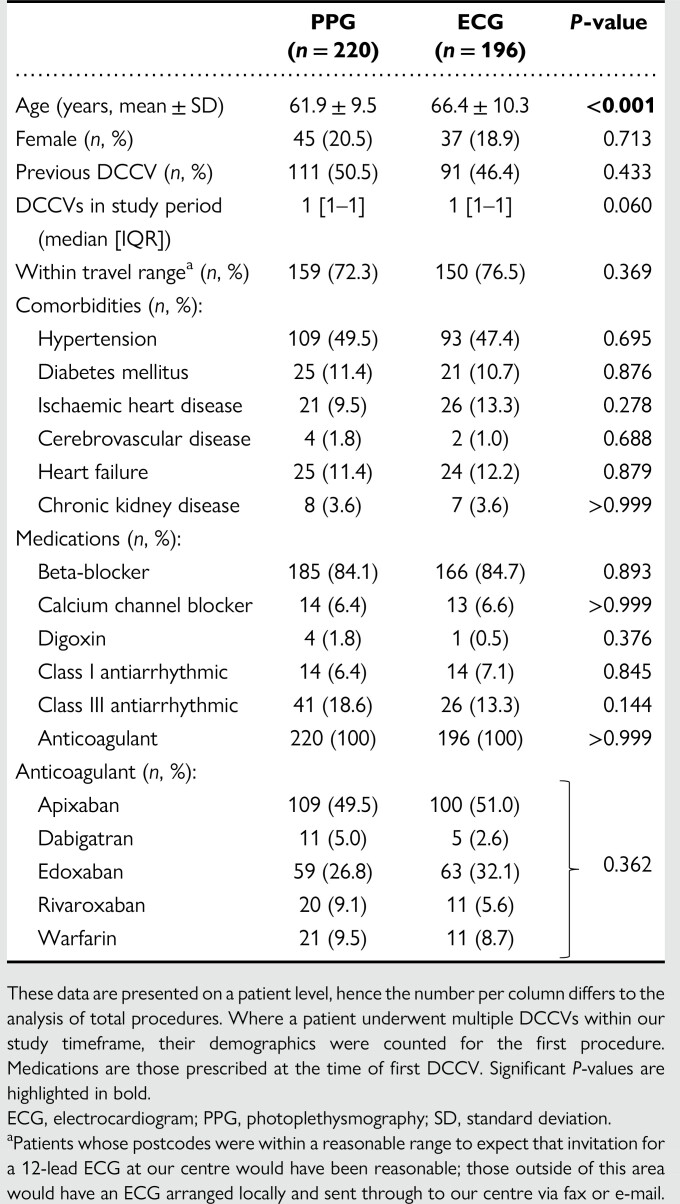
Demographics between patients undergoing PPG or ECG monitoring post-direct current cardioversion

The analyses below are assessed on a procedural level, rather than an individual patient level, in order to account for follow-up after each procedure.

### Uptake of photoplethysmography monitoring

*Table 2* shows a breakdown of reasons why PPG was not utilized in the 215 DCCV procedures followed up by ECG. In most cases, the reason for not utilizing remote PPG monitoring was unclear from electronic documentation. Anecdotally, we believe that the majority of these cases were due to patient preference. Where documented, the most common reasons included bradyarrhythmia and checking of the QT-interval due to antiarrhythmic drugs. Technical issues preventing assignment to the PPG arm were uncommon but included patients who did not own a smartphone (12 [5.6%]), inability to use the FibriCheck app (3 [1.4%]); or app technical failure (3 [1.4%]).

**Table 2:** Reasons for not utilizing FibriCheck following direct current cardioversion

	Number	Per cent
Not known^a^	84	39.3%
Bradyarrhythmia	49	22.9%
Check QT on antiarrhythmics	32	15.0%
Atrial flutter^b^	13	6.1%
No smartphone	12	5.6%
ECG coincided with clinic appointment	12	5.6%
Paced^b^	3	1.4%
Patient preference	3	1.4%
Patient unable to use FibriCheck	3	1.4%
FibriCheck technical issue	3	1.4%

^a^These cases utilized ECG follow-up but the reason why PPG monitoring was inappropriate was not clearly documented. Anecdotally, many of these cases were due to patient preference. We have since improved our documentation to capture these reasons more reliably.

^b^Atrial flutter and paced rhythms are regular and hence cannot reliably be distinguished from sinus rhythm using PPG. Most paced patients can have remote device interrogation, however some do not have this facility available and hence attended for an ECG instead.

### Rhythm at follow-up

Arrhythmia detection rates are shown in *Table 3*. These were generally similar between arms. Atrial flutter was more common in the ECG arm because patients with known flutter were preferred for ECG follow-up, since PPG cannot always reliably differentiate regular arrhythmias from sinus rhythm.

**Table 3:** Rhythm at follow-up

Rhythm (*n*, %)	PPG (*n* = 246)	ECG (*n* = 215)	*P*-value
Sinus rhythm	176 (71.5)	139 (64.7)	0.132
Atrial fibrillation	66 (26.8)	58 (27.0)	>0.999
Unknown^a^	1 (0.4)	4 (1.9)	0.189
Atrial flutter	2 (0.8)	8 (3.7)	0.051
Paced rhythm	1 (0.4)	5 (2.3)	0.102
Junctional rhythm	—	1 (0.5)	0.466

^a^Unknown refers to rhythms that are uncertain due to patient non-compliance with follow-up and lack of subsequent follow-up from which to determine their cardiac rhythm.

At short-term nurse specialist review, rate control medications were adjusted in 13.2% PPG vs. 19.7% ECG patients and antiarrhythmics were adjusted in 8.8% PPG and 7.6% ECG patients. In the remaining cases, no therapeutic changes were made. There was no statistical difference between groups (overall *P* = 0.583).

### Patient compliance and photoplethysmography technical failures

Overall, compliance with follow-up was high in both arms (PPG 89.4% vs. ECG 89.8%; *P* > 0.999).

Twenty-nine patients in the PPG arm (11.8%) subsequently required a 12-lead ECG. In 12 cases, this was due to the patient failing to download/utilize the app (non-compliance). In 10 cases, this was due to technical failure of the app. The remaining seven cases were due to the FibriCheck result being inconclusive. Of these seven, two ECGs showed AF, one showed atrial flutter and four showed sinus rhythm.

No patients in the ECG arm had a subsequent ECG arranged as part of DCCV follow-up—even in the event of non-compliance, as their follow-up clinic appointment tended to occur before another appointment could be made.

### Financial and environmental impact

Despite some PPG patients subsequently requiring an ECG, as described above, the mean cost to the healthcare system per patient was significantly lower in the PPG arm (£47.91 ± 43.62 vs. £135 ± 0; *P* < 0.001). Over the timeframe of our study (29 months), the total costs were £11 787 in the PPG arm vs. £29 025 in the ECG arm, or ∼£4871 vs. £11 994 per year, giving an annual healthcare saving of £7123.

Estimated median costs to the patient, including travel and parking, were £0 [0–0] vs. £5.13 [4.8–7.9] for PPG vs. ECG, respectively (*P* < 0.001). In total across our study timeframe, assignment to PPG monitoring cost patients a total of £177.27 compared with £1316.25 for ECG monitoring.

Median patient travel time (0 [0–0] min vs. 38 [30–71] min; *P* < 0.001) and carbon emissions (0 [0–0] kg vs. 2.32 [1.8–6.5] kg; *P* < 0.001) were significantly lower in the PPG arm. Across the study period, PPG patients spent an estimated total of 1240 min travelling, compared with 9935 min in the ECG arm. This resulted in a total estimated carbon emission of 110 kg in the PPG arm vs. 940 kg in the ECG arm.

### Safety

As per *Table 2*, 49 patients had an ECG for potential bradyarrhythmia. Of these, 31 (63%) had no concerning ECG findings. Seventeen (35%) had 1st degree atrioventricular block. One patient had a junctional rhythm and was admitted for pacemaker implant.

Thirty-two patients had an ECG for checking of the QT-interval. Of these, 28 (88%) had no concerning findings. One patient had a mildly prolonged corrected QT-interval (490 ms) and 3 patients had 1st degree atrioventricular block.

No adverse safety events were noted in the PPG arm.

### Patient experience of the FibriCheck application

**Figure 3:**
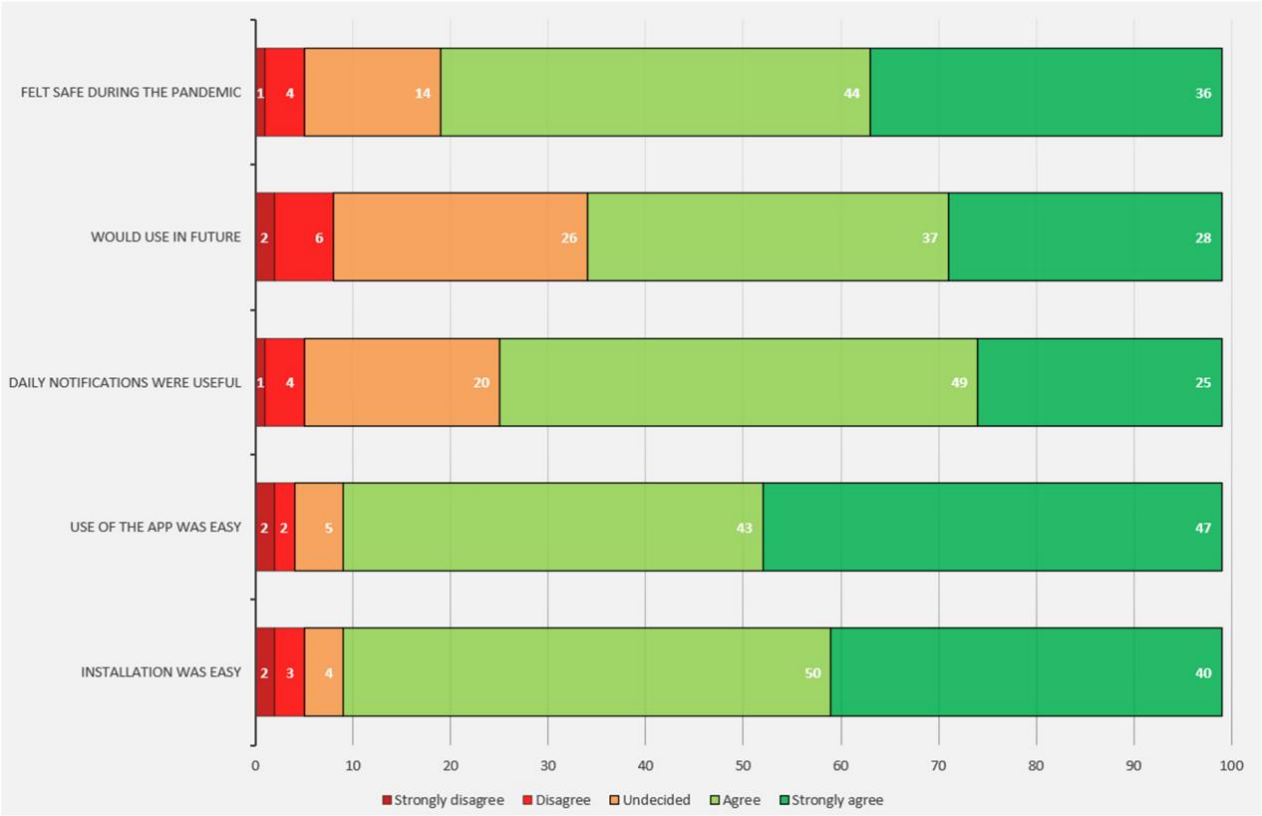
Patient responses to experience questionnaire regarding the use of the FibriCheck app at our centre (99 responses). These figures were provided by FibriCheck. Patients involved in the questionnaire were previously recruited at our site to the TeleCheck-AF study. The full results have been published as part of the larger study—these 99 responses were those from our centre alone and demonstrate overall satisfaction locally.

A total of 99 responses to the TeleCheck-AF patient survey were recorded at our centre. The results are summarized in *Figure 3*. The majority found both installation and use of the app straightforward. Similarly, most patients found the daily reminders helpful, though 20% were undecided. Most would use the app in the future, though 26% were undecided. Most also reported that the app was useful in the setting of the COVID-19 pandemic.

## Discussion

To the best of our knowledge, this is the first reported series of a real-world remote rhythm monitoring follow-up service using a smartphone PPG app after DCCV for AF. Our findings show that this approach is feasible and safe, acceptable to patients with good compliance, and significantly reduces costs to both the healthcare organization and patients. Patients also benefit from saving travel time, and CO_2_ emissions into the atmosphere are reduced.

It should be noted that, although FibriCheck provides daily reminders for patients to perform a PPG trace, most patients were advised to only take one reading at 14 days in order to replicate standard practice at the time. There may be additional benefits if the follow-up system can be adapted to account for daily readings. For example, this may allow earlier detection and management of AF recurrence or may detect paroxysmal recurrence of a previously persistent arrhythmia.

### Benefits of photoplethysmography monitoring

Photoplethysmography allows remote patient monitoring via a smartphone app, which is demonstrably easy to use and has been validated in prior studies.^9^ The TeleCheck-AF study demonstrated that FibriCheck is easily implemented within a rapid timeframe, even for centres inexperienced with mobile health technologies.^10^ This may be beneficial beyond DCCV, as part of a wider spectrum of mobile AF management.^9^

This approach was extremely helpful at our centre during the COVID-19 pandemic, permitting minimal patient contact during a high risk period of time. The AI algorithm and cloud-based software platform allowed for easy upload and review of data for both patient and clinician.

The FibriCheck dashboard, shown in *Figure 2*, is simple and easy to understand with minimal training required.

### Safety of remote monitoring

One of the major reasons for preferring a 12-lead ECG over remote PPG monitoring is for safety reasons, for example in patients where bradyarrhythmia or QT prolongation may be anticipated. Whilst our numbers were relatively low, only 1 of 32 patients with concern for bradyarrhythmia actually required admission for pacemaker implant due to junctional bradycardia, and no patients had a QTc above 500 ms. Hence, the vast majority of these patients could have been safely managed with PPG follow-up.

However, clinical safety remains critically important, thus we would not advise cessation of ECG-based follow-up. In particular, a bradycardic PPG trace in a patient with potential conduction system disease may simply prompt the clinician to order a 12-lead ECG, resulting in additional cost. Hence, where clinical concerns exist, a 12-lead ECG is perfectly reasonable as first-line follow-up.

### Cost, travel, and environmental benefits

Our data show significant benefits in terms of costs to the healthcare system and indeed to patients (Graphical Abstract). Patients also benefit from spending less time travelling to appointments, and avoid parking charges. This provides indirect benefits to other patients by freeing up parking spaces and reducing waiting times. The reduction in travel also has environmental benefits by reducing CO_2_ emissions.

Effective use of PPG monitoring is reliant upon patient adherence, and non-compliance can result in additional costs to the healthcare system. However, the cost is significantly less impactful with PPG monitoring compared with outpatient ECG appointments. A patient failing to attend an ECG appointment still costs the healthcare organization, and this is doubled if a further ECG appointment needs to be arranged. Conversely, if a patient fails to register for FibriCheck, a simple phone call may be all that is needed to prompt them to register and take a reading.

Although, in some cases, an ECG is required after PPG monitoring—either due to non-compliance, technical failure or poor quality PPG readings—our data show that this situation is infrequent (11.8% of the PPG arm) and that, overall, there is a significant net cost saving with PPG-based follow-up.

Additionally, FibriCheck costs £32 per activation per year, so further savings may be expected in those patients who undergo more than one cardioversion within the same year. Furthermore, ECG appointments negated by PPG monitoring can be utilized for other patients, providing additional benefits.

### Expanded uses of photoplethysmography

As alluded to above, it may be possible to allow earlier detection of recurrent AF following DCCV by regular monitoring of daily recordings. This could also be expanded to other settings, such as post-catheter ablation, where early recurrence may portend a greater risk of long-term recurrence.^14^

Furthermore, in the emergency department (ED) setting, it has been shown that AF may spontaneously resolve in over two-thirds of cases within 48 h.^15^ Where patients and clinicians prefer this ‘watch-and-wait’ strategy, PPG may be a useful adjunct, as proposed in the TeleWAS-AF study.^16^ This may improve management and reduce the burden of new-onset AF on the ED system.

Our findings suggest that PPG monitoring could be utilized more frequently—i.e. as first-line follow-up following DCCV and in preference to a 12-lead ECG—in most cases. It is important to consider the risk of rare adverse outcomes, however—for example, one patient required urgent pacemaker implant. In this sense, we consider individualized decision making with clinical judgement, erring on the side of caution, the optimal approach.

### Limitations

Our study describes a single centre experience using FibriCheck, hence our findings may not be generalizable to all patients undergoing DCCV for AF, nor to other apps or different arrhythmias. There was an imbalance in patient age between the analysed groups, which may induce some bias in the results; however the cohorts were otherwise well-balanced. Cost savings may not be generalizable to all healthcare systems in all countries.

Photoplethysmography monitoring is subject to some limitations. Regular arrhythmias, such as atrial flutter, are challenging to detect via PPG. Similarly, bradycardia may be detected but the underlying mechanism cannot be assessed. This may be improved upon by applying a stepwise interpretation approach as described in the TeleCheck-AF PPG dictionary^17^ and by optimizing device usage as per the European Heart Rhythm Association practical guide.^18^ However, in some cases, a 12-lead ECG will still be required, as discussed above.

Some patients may not have compatible smartphones or may be unable to operate the app due to, for example, cognitive impairment of movement disorders. This emphasizes the importance of appropriate patient selection.

## Conclusion

Remote PPG monitoring via a smartphone app is highly effective in avoiding unnecessary patient re-attendance following DCCV, and provides significant cost, time, and environmental savings.

## Data Availability

In order to maintain privacy, the data underlying this article cannot be made publicly available. Anonymised data may be made available upon request.
